# Nutritional Transition in Children under Five Years and Women of Reproductive Age: A 15-Years Trend Analysis in Peru

**DOI:** 10.1371/journal.pone.0092550

**Published:** 2014-03-18

**Authors:** Christian Loret de Mola, Renato Quispe, Giancarlo A. Valle, Julio A. Poterico

**Affiliations:** 1 CRONICAS Center of Excellence in Chronic Diseases, Universidad Peruana Cayetano Heredia, Lima, Peru; 2 Post-Graduate Program in Epidemiology, Federal University of Pelotas, Pelotas, Rio Grande do Sul, Brazil; 3 CONEVID Unidad de Conocimiento y Evidencia, Universidad Peruana Cayetano Heredia, Lima, Peru; US Naval Medical Research Center Detachment Research Unit 6, Peru

## Abstract

**Background:**

Rapid urbanization, increase in food availability, and changes in diet and lifestyle patterns have been changing nutritional profiles in developing nations. We aimed to describe nutritional changes in children under 5 years and women of reproductive age in Peru, during a 15-year period of rapid economic development and social policy enhancement.

**Materials and Methods:**

Trend analyses of anthropometric measures in children of preschool age and women between 15–49 years, using the Peruvian National Demographic and Family Health Surveys (DHS) from 1996 to 2011. WHO growth curves were used to define stunting, underweight, wasting and overweight in children <5y. We employed the WHO BMI-age standardized curves for teenagers between 15–19y. In women >19 years, body mass index (BMI) was analyzed both categorically and as a continuous variable. To statistically analyze the trends, we used regression models: Linear and Poisson for continuous and binary outcomes, respectively.

**Results:**

We analyzed data from 123 642 women and 64 135 children, from 1996 to 2011. Decreases over time were evidenced for underweight (p<0.001), wasting (p<0.001), and stunting (p<0.001) in children under 5y. This effect was particularly noted in urban settings. Overweight levels in children reduced (p<0.001), however this reduction stopped, in urban settings, since 2005 (∼12%). Anemia decreased in children and women (p<0.001); with higher reduction in urban (↓43%) than in rural children (↓24%). BMI in women aged 15–19 years increased (p<0.001) across time, with noticeable BMI-curve shift in women older than 30 years. Moreover, obesity doubled during this period in women more than 19y.

**Conclusion:**

Nutrition transition in Peru shows different patterns for urban and rural populations. Public policies should emphasize targeting both malnutrition conditions—undernutrition/stunting, overweight/obesity and anemia—considering age and place of residence in rapid developing societies like Peru.

## Introduction

Since the 1960s, worldwide economic growth, has been accompanied by an increase in food availability, animal fat intake, less physical activity and urbanization [1]. This nutritional transition has been changing anthropometric and health patterns throughout populations, having special impact in low and middle-income countries (LMIC) [2].

Underweight and stunting have been dropping in LMIC, mainly in children under 5 years [3]; but overweight depicts diverse distribution and rates between children populations [3], [4]. Since 1990, overweight rates have been raising in children from high and low-income countries. However, children from some upper-middle income societies depict a slight decrease in over-nutritional indicators, suggesting a diverse nutritional transition in children of preschool age [3]; and that country's development and economic growth may play an important role.

In the last 30 years, body mass index (BMI) in adults has been increasing in the world [5], and during this period global age-standardized obesity prevalence has nearly doubled [6] and developing countries appear to be at the forefront of this trend [7], [8], and rapid nutritional changes in urban settings might explain the over-nutrition climb [2], [7].

Previous studies have reported nutritional characteristics and trends of Peruvian children and adults through population-based datasets. For instance, a recent publication reported a high prevalence of over-nutrition (overweight or obesity) in Peruvians between 30–59 years (62.3%), where women had higher rates compared to males [9]. From 1991 to 2005, stunting decreased but overweight increased in children of preschool age [10]. Similar analyses from 2007 to 2010 showed that overweight in children under five years was related to: first year of life, male gender, birth weight more than 2.5 kg and living in high urbanized areas (i.e. Lima and the Coast region) [11]. However, these studies lacked of nutrition characterization during the entire period of economic development and social policy enhancement in Peru.

Anemia represents another malnutrition indicator in children and women. Global estimates show a concentration of this condition in low socioeconomic groups, and anemia in children correlates strongly to maternal anemia [12]. In Peru, factors associated with anemia in children of preschool age are: lack of prenatal care, adolescent mother and poor socioeconomic position [13].

Peru, as other Latin-American countries, has experienced a rapid economic growth in the last two decades. According to the World Bank, in 1996, Peru had a Gross Domestic Product (GDP) of $55.9 billion (current $) and a Gross National Income (GNI) per capita of $2200; whereas, in 2011, GDP was reported as $176.9 billion and GNI per capita reached to $5150 (http://data.worldbank.org/country/peru). Since 2006, social programs directed to aiding poor populations have enhanced nutritional programs in Peru [14]. Information of the Peruvian situation would help to better understand how combined economic growth with social policy targeting could affect nutritional transition in rapid developing nations.

We aimed to analyze in this study the nutritional transition of the Peruvian population through anthropometric measures—and anemia levels—of children under 5 years and women in childbearing age; using Peruvian Demographic Health Surveys during a 15-year period and stratifying analyses by urban and rural settings.

## Methods

### Ethics Statement

During data collection, INEI was responsible for obtaining an informed consent from all participants for the survey information, anthropometric measures and hemoglobin test. Using an information sheet, the research team explained to the participants the nature of the study and what kind of question they were going to answer as well as what kind of measure they were going to take. For survey and anthropometric data, verbal informed consent was obtained for all women, including adolescents, which, in addition, required their parents' or legal guardian's consent. For children under five years, verbal consent was obtained directly from their parents or legal guardians. For hemoglobin tests a written informed consent was required and in the case of adolescents and children under 5 years, their parents or legal guardian had to sign the consent and the adolescent give their assent. The consent forms stated that this was a national survey sponsored by the Peruvian Government and explaining its nature and importance, that all collected information would be confidential and that the person could stop answering the questions or reject to continue participating in this research at any time, explained the kind of questions they were going to answer, the measures that were going to be taken and the time this survey would take, depending on the amount of children under 5 and women in reproductive age living in the house, it also explained the nature and procedures they would use for the hemoglobin test, including a detailed explanation, which included showing the materials they were going to use for the test, as well as explaining that only one drop of blood was going to be taken from them, in addition it explained to them what anemia was, its signs and symptoms. The acceptance or rejection to answer the survey, give their anthropometric measures or do the hemoglobin test, was documented by the field worker in the response sheet. ENDES methodological procedures were reviewed and approved by the IRB from the Peruvian National Institute of Health and the “Centro de Alimentacion y Nutricion” (CENAN). In addition, the study protocol, specifically for this secondary analysis, was sent to the ethics committee of the Universidad Peruana Cayetano Heredia. They considered that this secondary analysis and the original research did not violate any ethics procedure, and approved further analysis and publication (SIDISI Code 61753).

### Study Design

Secondary analysis, evaluating the trends of anthropometric measures in children under 5 years and women of reproductive age using the Peruvian National Demographic and Family Health Surveys (DHS), from 1996 to 2011. We also included percentages of anemia as other malnutrition indicator in both children and women.

DHS were cross-sectional surveys performed by the National Institute of Statistics and Informatics (INEI) in Peru; each being a nationally representative, multi-staged, and probabilistic study. Each wave included participants from rural and urban areas. These surveys formed part of the Monitoring and Evaluation to Assess and Use Results Demographic and Health Surveys (MEASURE DHS), until 2008. Since then, the Peruvian government and INEI have supported, conducted and monitored the surveys—using similar methodologies used in previous DHS (http://www.measuredhs.com/Data/).

### Participants and Settings

Each DHS recruited women between 15 to 49 years and children aged 5 years or younger who lived in the same household. In rural areas, villages of 500–2,000 people were the primary sampling units, and households within each of these clusters were the secondary sampling units. In urban areas, primary sampling units consisted of blocks or groups of blocks with more than 2,000 people (with 100 houses on average), and secondary sampling units were the same as in rural settings. More details about the methodology for each survey are available publicly (http://desa.inei.gob.pe/endes/).

For some waves, the total sampling goal was achieved in more than one year (namely continuous DHS), but the annual-based sample remained representative of the Peruvian population in that specific year. We used all available surveys from 1996 to 2011, having eight waves: 1996, 2000, 2004–2006 (which only had children anthropometric data for 2005), 2007–2008, and 2009–2011. Household response rates, were above 93% in all surveys.

For this analysis, we included all children under 5 years and non-pregnant women with anthropometric and hemoglobin information. We had available data for 93.1% of children <5y and 64.7% of all interviewed women. Anthropometry in 1996 was measured in a subsample of women (29.8%), afterwards, 90% of non-pregnant women had available data.

### Measurements and Variables

Anthropometric measures were recorded by trained personnel in all eight waves (1996, 2000, 2005, 2007, 2008, 2009, 2010, and 2011). Participants' weight was measured using solar-powered mother-baby scales with an accuracy of ±100 g. Since 2007, an electric scale (SECA model 881-872) has been used, having a maximum load of 150 kg. Previously, a similar scale (SECA model 874) was employed, which had a maximum load of 200 kg. Height was evaluated using a wooden stadiometer—an adjustable board calibrated in millimeters—following international standards for DHS [15]; measuring adults and children older than 2 years in a standing position, whereas children younger than 2 years lying down.

We evaluated three under-nutritional variables in children under 5 years: stunting, wasting, and underweight. We defined these indicators as: having a height for age, weight for height, and weight for age in Z score less than 2 standard deviations (SD) below the mean of the World Health Organization (WHO) international reference standards, respectively. Overweight (including obesity) in children <5y was defined as having a BMI-for-age more than 2 SD above the mean of the WHO international standards [16].

In women between 19 and 49 years, nutritional status was based on BMI, which was subsequently analyzed as a continuous and categorical variable. Using WHO standards, we used the following definitions in women older than 19y: underweight (BMI≤18.5 kg/m^2^), normal (18.5-<25 kg/m^2^), overweight (25-<30 kg/m^2^), and obese (≥30 kg/m^2^) [17]. Available information of these definitions can be found at: http://apps.who.int/bmi/index.jsp. For women between 15 and 19 years, we used WHO standardized growth curves because BMI cut-off points for obesity and overweight in this age range may differ from adults. Thus, we defined a BMI-for-age of more than 1 SD but less than 2 SD above the mean as overweight, and more than 2 SD as obese [18].

We also evaluated trends in women and children socio-demographic variables: age, years of education, child sex and place of residence—all information was asked to children's mothers. Anemia was defined using WHO cut-offs for non-pregnant women (hemoglobin count <12 g/dl) and children aged 5 to 59 months(hemoglobin count<11 g/dl)[19]. We used years of education as a proxy for socioeconomic status (SES) because the wealth and assets indexes constructed by INEI did not maintain standard variables across waves, making comparability and standardization difficult. In addition, it has been shown that for rural populations' assets index are not a good SES indicator [20], so for our analysis we used women education as a proxy of SES.

### Statistical Analysis

Descriptive analyses were conducted for all DHS waves calculating prevalence and mean values for the evaluated variables, as appropriate. Moreover, we plotted women's BMI curves of 1996 and 2011 to compare general BMI changes—stratifying by age range (15–19; 20–29; 30–39; 40–49). We divided women in age-groups due to different BMI shifts by age. Our intention was to show the secular trend in BMI and women's age temporary effect modification. Children BMI curves standardized for age and sex were plotted according to WHO growth curves [21], in urban and rural comparing 1996 and 2011.

We performed trend analysis, stratifying by place of residence (urban or rural), using linear regression models for continuous variables (e.g. BMI); and Poisson regression for binary outcomes calculating prevalence ratios (PR) [22]. We analyzed every anthropometric variable independently in a regression model: each category of women's BMI (Poisson regression), continuous BMI (linear regression), each nutritional variable in children (Poisson regression), anemia (Poisson regression), and socio-demographic variables.

The year of survey was employed as a continuous independent variable, evaluating any linear increase or decrease across years. When data showed a visual non-linear trend, we analyzed total rise or decrease using the survey-year as a dummy variable. When this happened, we tried to analyze smaller periods in which trends appeared to be linear. For instance, if we evidenced a linear decline from 1996 to 2005, but a flattened pattern since then or inverse direction of the trend, we analyzed separately the periods 1996–2005 and 2005–2011.

We also compared in some cases differences in point prevalence (for any specific year) between urban versus rural populations using regression models (Poisson). Since BMI cut-off points were different for adult (20–49 years) and adolescent (15–19 years) women, we performed separated analyses to evaluate trends of categorized BMI (obesity, overweight, normal, underweight). Adolescents' analyses were not stratified by place of residence due to small sample sizes in some periods.

STATA version 11.2 software (STATA Corp, College Station, TX, US) was employed for our analyses. Given surveys' multistage random samples, we used the *svy* set of commands. Accounting sampling units and stratification of the samples, we calculated weights of the analyzed individuals in the household and linearized standard errors (SE). We calculated 95% confidence intervals and p<0.05 was considered significant in our analyses.

## Results

We analyzed data from 123,642 women—78,408 urban and 45,234 rural—and 64,135 children under 5 years—33,304 urban and 30,831 rural—in all DHS waves (1996 to 2011). Table 1 shows a detailed description of populations' socio-demographic characteristics and sample size for each wave.

**Table 1:** Age, education, anemia, sex and BMI description of Women and children under five years old in Urban and Rural Areas of Peru between years 1996–2011.

	Urban								Rural							
	Mean/Proportion								Mean/Proportion							
	*1996*	*2000*	*2005*	*2007*	*2008*	*2009*	*2010*	*2011*	*1996*	*2000*	*2005*	*2007*	*2008*	*2009*	*2010*	*2011*
***Women***	6,128	15,715	3,479	3,101	9,121	13,842	13,520	13,502	4,715	9,791	2,198	2,129	4,073	7,334	7,509	7,485
***Age*** *	29.5	29.5	30.0	30.4	30.4	30.3	30.5	30.5	29.6	29.8	30.5	30.6	31.1	30.7	30.7	30.9
***Education*** *	8.4	10.2	10.9	10.7	10.6	10.6	10.7	10.8	4.0	5.8	6.1	6.4	6.3	6.4	6.5	6.5
***BMI***	25.61	26.15	25.59	26.22	26.31	26.35	26.40	26.97	24.27	24.55	24.69	24.96	24.92	25.09	25.07	25.29
***Anemia*** **	ND	28.9%	27.1%	27.2%	25.2%	20.7%	20.1%	16.5%	ND	36.6%	31.2%	30.0%	25.4%	22.0%	24.7%	19.0%
***Children***	7938	5337	1066	1086	3257	5115	4731	4774	7171	6384	1248	1308	2465	4257	4038	3960
***Male***	50.5%	50.5%	51.4%	50.1%	49.7%	51.2%	50.5%	51.2%	50.3%	51.1%	51.8%	49.7%	50.3%	52.2%	49.2%	49.1%
***Age*** *	2.5	2.5	2.4	2.5	2.5	2.5	2.5	2.5	2.4	2.5	2.5	2.5	2.6	2.5	2.5	2.5
***Anemia*** **	ND	45.8%	ND	37.2%	39.9%	32.8%	33.2%	25.9%	ND	52.3%	ND	49.6%	47.2%	43.7%	45.9%	40.0%

ND = No Data.

*Mean of years of education and age.

**Percentage of anemic children/women.

Overall, 73% of women were from urban areas maintaining this percentage throughout years (p = 0.132). Women's mean age, across waves, was 30.3 years SE[0.03]; with 19.7% from 15 to 19 years old, 30.5% from 20 to 29 years, 27.3% from 30 to 39 years, and 22% from 40 to 49 years. Mean years of education in women increased in both settings (p<0.001), especially from 1996 to 2000 with a greater upsurge among urban women over time. Anemia among women declined significantly from 1996 to 2011 (p<0.001), with similar reductions in rural (decrease of 48% SE [2.7]) and urban (decrease of 43% SE [2.8]) areas. In 2011, we found that rural women had higher prevalence of anemia compared to their urban counterparts (p = 0.005) (Table 1). Conversely, children from urban areas (decrease of 43%, from 1996 to 2011) depicted a higher anemia diminution compared to rural counterparts (decrease of 24%, from 1996 to 2011, p<0.001).

Children's mean age, across waves, was 2.5 years SE [0.01] with similar values across all waves and no differences between urban and rural areas. The number of evaluated male and female children across time was constant (∼1∶1 proportion). A declining trend of anemia in children was observed in both settings (p<0.001). Comparing 2000 to 2011, children with anemia in the overall analysis had a 48% reduction (PR = 0.62 SE [0.02]), decreasing more in urban (PR = 0.57 SE [0.03]) than in rural settings (PR = 0.73 SE [0.03]). Moreover, the prevalence of anemia among rural children was 46% higher than in their urban counterparts (p<0.001) in 2011 (Table 1).

### Nutritional Trends for Children

**Figure 1:**
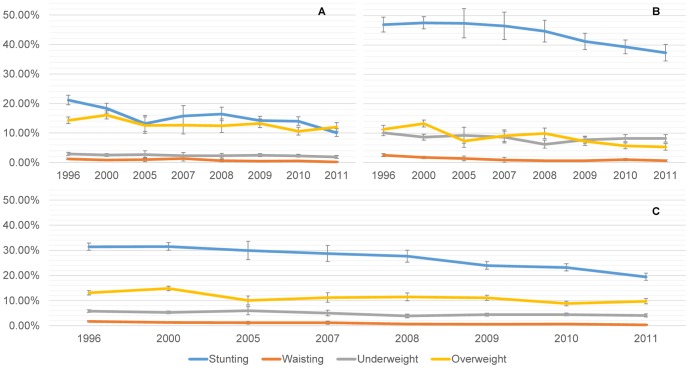
Stunting, wasting, underweight, and overweight trends of urban and rural children under five years, 1996–2011. A. Urban children (n = 33,304). B. Rural Children (n = 30,831). C. All Children (n = 64,135).

Between 1996 and 2011, stunting, wasting, and underweight decreased (p<0.001 for all three). A small decrease in overweight prevalence was also observed (p<0.001) (Figure 1).

Stratifying by place of residence, we observe a deeper decline of stunting in urban (51.5%) than rural (19.6%) areas (p<0.001) over time. Figure 1 shows that stunting decrease started in 2007 for rural children, having a significant reduction from 2007 to 2011 (p<0.001), but not from 1996 to 2007 (p = 0.973).

During the analyzed period, wasting declined in urban (p<0.001) and rural (p<0.001) children, with no significant reductions from 2007 to 2011 in the rural areas (p = 0.979). In 2011, wasting prevalence was 0.22% SE [0.08] and 0.64% SE [0.02] in urban and rural children, respectively. On the other hand, underweight has also been dropping in both settings, having a constant reduction in urban children, going from 2.9% to 1.9% (p<0.001). In the rural children, underweight dropped from 10.1% to 8.2% in an irregular way (p<0.001) (Figure 1).

In urban children, overweight prevalence declined from 1996 to 2011 (p<0.001), but this trend seemed to have flattened since 2005 (p = 0.235); depicting in 2011 an overweight prevalence of 12.0% SE [0.73], surpassing stunting levels (10.3% SE [0.66]). In rural children, overweight declined between 1996 and 2011 (p<0.001).

### Women Nutritional Trends

**Figure 2:**
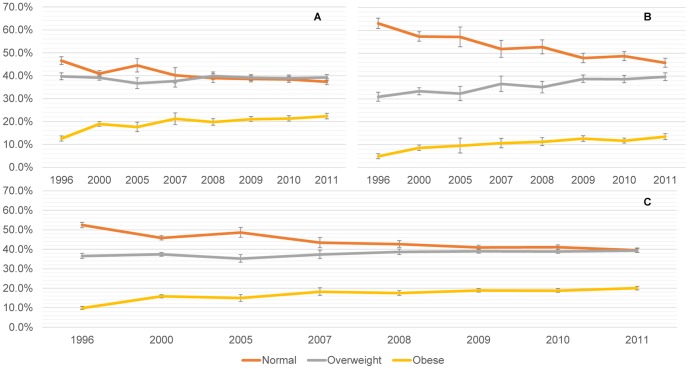
Categorical BMI trends for Peruvian urban and rural adult women (20–49 years), 1996–2011. A. Urban adult women (n = 63,895). B. Rural adult women (n = 37,088). C. All adult women (20–49 years) (n = 100,983).

We observe an overall increase in the prevalence of overweight from 1996 to 2011 (p<0.001), in women between 20–49 years. However, the curve flattened since 2008. Percentages of women with normal BMI have been declining over time (p<0.001), especially since 2005; showing similar percentages of normal (39.6% SE [0.56]) and overweight (39.4% SE [0.52]) women in 2011 (Figure 2).

Obesity levels depict a constant increase over time (p<0.001). In 2011, the prevalence of overweight/obese women was higher than the normal BMI-category (p<0.001); and underweight maintained constant across years with a prevalence of ∼1% in most waves for urban and rural settings.

Overweight did not show a clear trend in urban women (p = 0.796), but percentages of normal women declined, especially since 2005 (p<0.001). We evidenced for 2011 similar percentages of overweight (39.3% SE [0.63]) and normal-BMI women (37.4% SE [0.67]) (Figure 2). Furthermore, obesity increased 1.78 (SE [0.09]) times in 2011 compared to 1996 (p<0.001) (increase of 0.65% points per/year). In 2011, 22.3% adult women (SE [0.06]) were obese, and 61.6% SE [1.03] had a BMI ≥25 kg/m^2^.

Rural areas depicts a decrease of normal-BMI women (p<0.001), meanwhile overweight prevalence increased over time (p<0.001), specially since 2005 (Figure 2). Obesity shows a constant increase during the analyzed period (p<0.001) and its prevalence in 2011 was 2.75 (SE [0.35]) times higher than in 1996. On the other hand, for rural women in 2011, the prevalence of normal-BMI (45.9%) was higher than overweight (39.7%) women (p<0.001); but lower than obese/overweight females (53.2%) (p<0.001).

**Figure 3:**
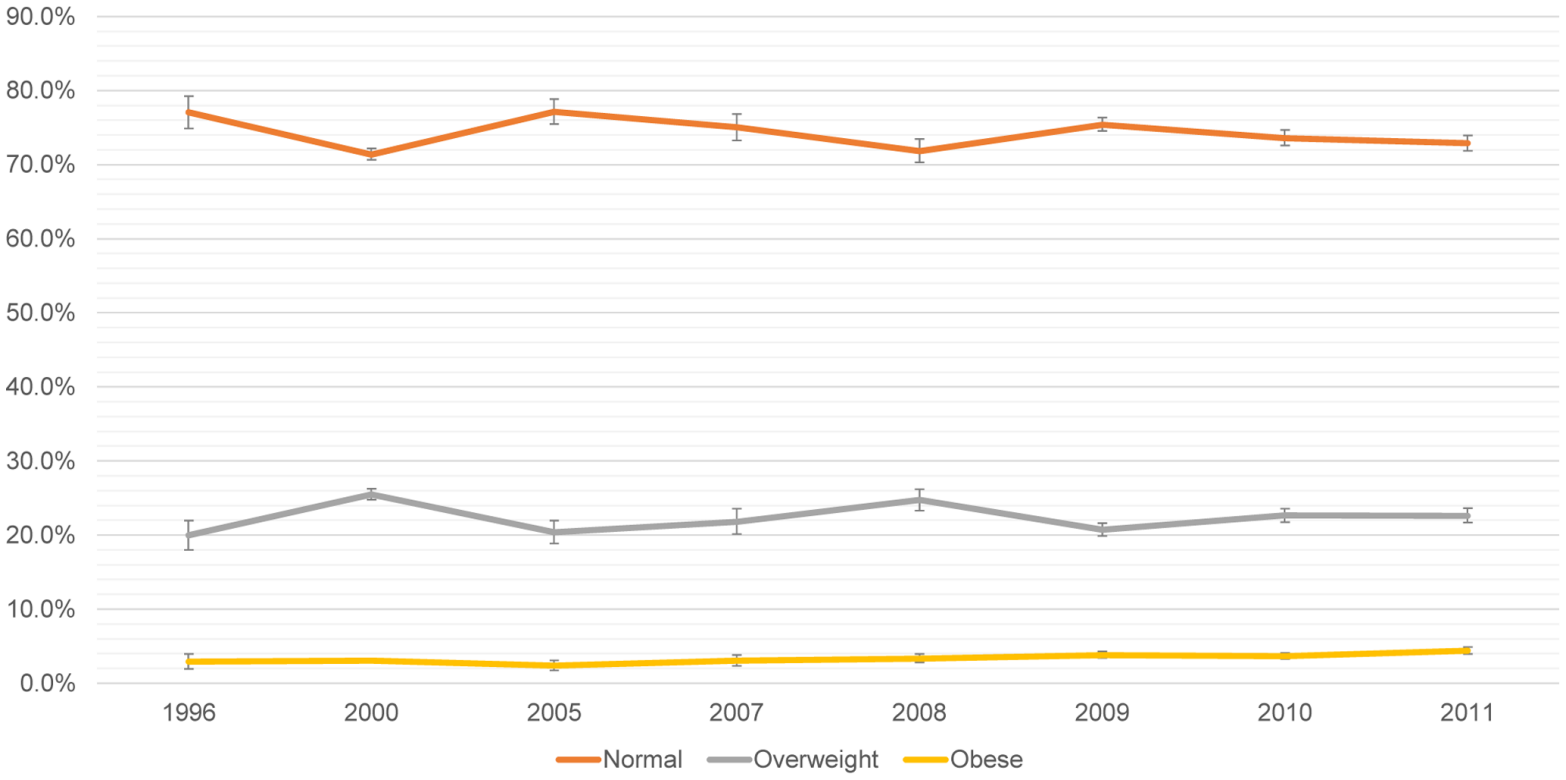
Categorical BMI trends for Peruvian adolescent women (15–19 years), 1996–2011. Total n = 22,659; 1996 n = 717; 2000 n = 5,340; 2005 n = 1,154; 2007 n = 1,001; 2008 n = 2,462; 2009 n = 4,021; 2010 n = 4,038; 2011 n = 3,926.

Teenagers between 15 to 19 years, showed a slight increment in obesity since 2005 (p = 0.020) (Figure 3).

In Table 1, we observe that women's mean BMI has been constantly increasing from 1996 to 2011 (p<0.001): ↑0.09 kg/m^2^ per year. Mean BMI augmented for urban and rural women (p<0.001 in both settings), with a higher increase per year in urban (↑0.19%/year SE [0.04]) than rural (↑0.09%/year SE [0.04]) settings.

### BMI Curves

**Figure 4:**
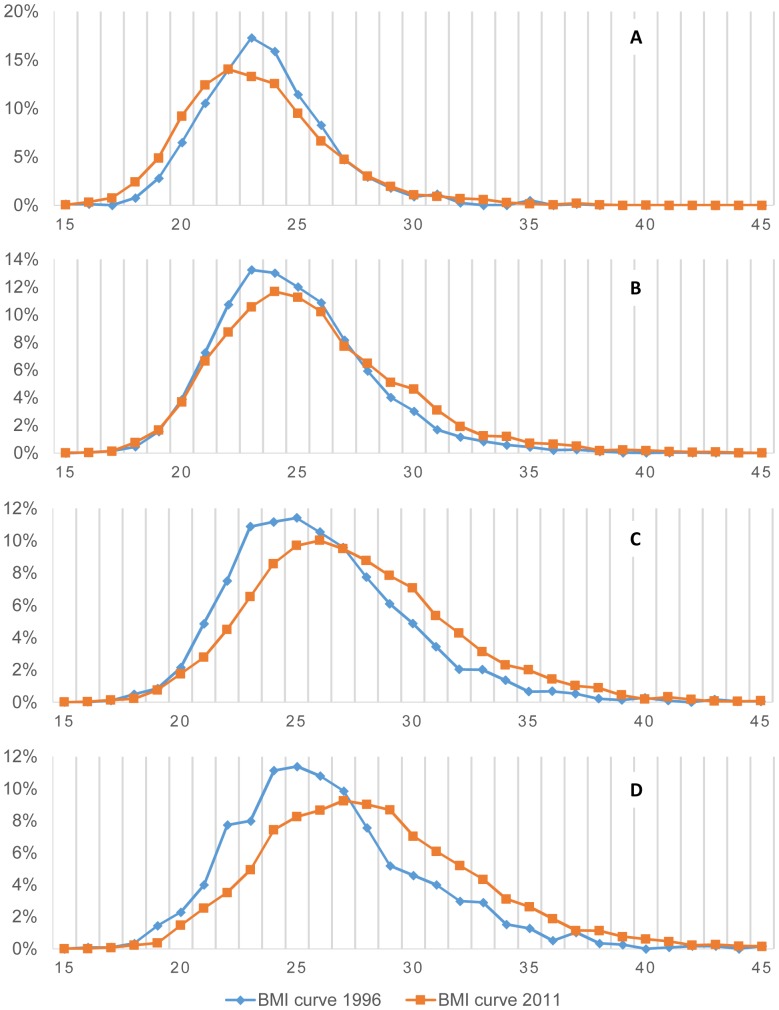
Peruvian women BMI curves, 1996–2011, stratified by age. A. 15–19 years (1996 n = 717/2011 n = 3,926) **B**. 20–29 years (1996 n = 5,141/2011 n = 6,058) **C**. 30–39 years (1996 n = 3,836/2011 n = 5,928) **D**. 40–49 years (1996 n = 1,149/2011 n = 5,075). Y-axis Percentage of women with a given BMI in each age group; X-axis Women BMI.

In figure 4, when comparing women's BMI curves of 1996 and 2011, we can see a slight shift to the right and flattening of the curve in 2011 in adult women between 30–39 and 40–49 years. When looking only at the curves from 1996, data suggests that older populations had very little increase in overall BMI. However, in 2011 this changed, and we evidence that older populations tend to increase their overall BMI more than in 1996; consequently, women from 1996 increased their BMI in 2011 more than expected. Women between 20–30 years in 1996 and 2011 did not have differences in BMI curves, however, those from 1996, which would be in the 30–40/40–49 age groups in 2011 had more flattened BMI curves with an evident shift to the right, when compared with the same age groups from 1996. Suggesting, therefore, a temporary modification of the effect of age on the risk of BMI increase.

**Figure 5:**
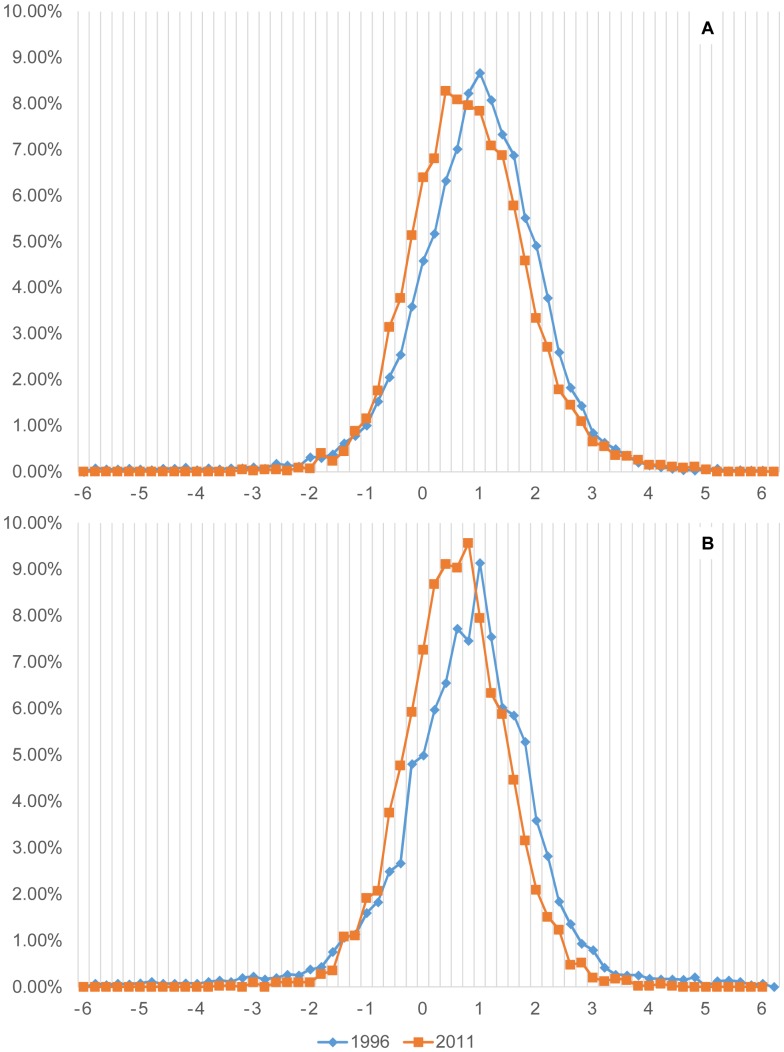
Peruvian Children BMI curves standardized for age and sex using WHO reference, 1996–2011. A. Urban Children (1996 n = 7,938/2011 n = 4,774) **B**. Rural children (1996 n = 7,171/2011 n = 3,960). Y-axis Percentage of children with a given BMI; X-axis WHO age-sex Standardized BMI.

We plotted the BMI distribution for children under 5y, noticing that rural and urban children's curves maintained constant between1996 to 2011 (Figure 5).

## Discussion

Peru has experienced a rapid nutritional transition in a short period of time with remaining malnutrition associated with stunting and anemia; and increasing rates of obesity in adult women.

We found declining levels of chronic malnutrition (stunting) in children under 5 years since 2007, with greater reduction in urban (↓51.5%) than rural (↓19.6%) areas from 1996 to 2011; with similar patterns of wasting and underweight. Overweight in children have declined in both rural and urban settings, but since 2005, the overall decrease levelled off.

Women's mean BMI increased over time, especially in females older than 30 years. In addition, not just the overall BMI of adult women augmented, but also the risk of increasing their BMI with older age.

Furthermore, obesity doubled during this15 year-period in both urban and rural adult women. Anthropometric profile of rural women in 2011 resembled to what urban counterparts presented in 1996; reflecting a 15-year nutritional gap between these two settings.

The prevalence of anemia has been decreasing in Peruvian women in both settings from 2000 to 2011; with greater reduction in rural women (↓43%). Conversely, anemia reduced more in urban children (↓48%); maintaining high levels of anemia in rural areas.

Similarities in proportions of urban women and mean age across the studied waves, shown in table 1, suggests that sample populations were comparable and trends are not an artifact of sampling.

Children from other developing countries have shown decreasing levels of chronic malnutrition [23]; and underweight [3]. In Peru, Mispireta *et al* [10] reported slight decreasing stunting levels, from 38.9% to 31.7% during 1991 to 2005. They also found that stunting prevalence in Peruvian rural areas remained high, but urban areas evidenced a more prominent reduction. These findings agree with ours, but our analysis included later years and we were able to demonstrate that chronic malnutrition reduction was greater beyond 2005.

Social factors enhancement might have contributed to reduce chronic malnutrition levels in Peru, such as: reduction in maternal mortality [24], increasing access to water and sanitation [13], [25], and improvement of breastfeeding rates [13] and education in women. The latter statement could support any beneficial effect of mother's education in chronic malnutrition in children under five years between 1996 and 2011. We show in this period a 27% and 62.5% increase in the mean of years of education for urban and rural women, respectively. Other Latin American countries have reduced their stunting levels improving maternal schooling, health care, sanitation, purchasing power of families [26]; and fortified supplements and cash transfer interventions [27]. Thus, improving Peruvian families living conditions might have been associated with reductions in stunting levels through social programs [14].

Peru has experienced a particular scenario regarding its nutritional and social programs, which may explain declining stunting levels after 2005 reported in this manuscript.

It seemed that Peruvian feeding programs had not being contributing to chronic malnutrition levels until 2005 [14]. Therefore, the Peruvian Government with other national and international institutions joined to form in 2006 the Children Malnutrition Initiative (CMI), placing nutrition in the priority agenda. With CMI, strong political commitment and technical support helped to struggle against stunting. Social programs such as CRECER (National Strategy for Poverty Reduction and Economic Opportunities, implemented in 2007) and JUNTOS (the Government's target cash transfer program) may have improved stunting levels in a short period—since [14]. The former program focused on children and pregnant women in poor areas; and the latter targeted on the reduction of children malnutrition and increase in families' use of children and maternal health care services. Thus, social policy enhancement seems to play a role in the fight against stunting.

Acosta [14] suggested that socioeconomic growth and the commodities boom did not affect stunting level, but poverty reduction did. Despite the Peruvian development, social programs have faced challenges in rural areas until more recently in mid 2000's because of internal conflict (i.e. terrorism) in 1980's and early 1990's. These challenges might have maintained stunting disparities between urban and rural areas, supported by our results. Between 1996 and 2011, social and nutritional programs intensified rural malnutrition targeting; and enhanced of social programs focused in vulnerable groups after 2007 [14].

Education improvement in mothers might have affected levels of children with overweight in Peru, because more educated women could have less overweight children [28]; and nutritional programs and subsidized meals in low income populations might have exerted an unintentional impact in overweight of children under 5y [29]. Our declining pattern in overweight in children under 5y resemble to what WHO-UNICEF-World Bank have reported since 1990 in upper-middle income countries [3]. Although overweight and obesity levels in preschool children of some Latin American countries appear to be low [30], de Onis *et al* have reported an overall increase in this region from 1990 to 2010 [21]. Moreover, augmenting rates in LMIC are projected [21], mainly affecting urban areas and privileged groups [9], [31].

Several factors for malnutrition control have been reported in rapid-growing societies of the Latin American region. For instance, in Brazil, 4 factors contributed with two-thirds of stunting decrease between 1996 to 2007: 25.7% to improved maternal schooling; 21.7% to an increase in the purchasing power of families; 11.6% to the expansion of health care; and 4.3% to improved sanitation [26]. Meanwhile, Mexico has been succeeding the fight against stunting with supplemented fortified supplements, health-care, education and cash transfer interventions [27].

Peruvian women have increased their BMI in 1.3 kg/m^2^ between 1996 and 2011 (with a rate of 0.09 kg/m^2^ per year), surpassing—and almost doubling—global women trend rate reported by Finucane *et al*. [5]; but supporting the fact that Latin American countries are facing a rapid nutritional transition. Worldwide, adult BMI has increased: 0.4 kg/m^2^ and 0.5 kg/m^2^ per decade in males and females, respectively, from 1980 to 2008, with Latin America presenting the second highest BMI rise after Oceania [5]. Moreover, adult global age-standardized obesity and overweight levels have augmented, from 6.4% to 12.0%, and from 24.6% to 34.4%, respectively [6].

Mendez *et al*. showed that more urbanized and developed LMIC tend to have higher levels of over-nutrition, and women of most developing societies reflect more overweight than underweight [7]. In a previous study with nationally representative data from Peruvian women of reproductive age, over-nutrition reached 44.7% in 2005, with concomitant raising levels of obesity from 1991 to 2005 (8,9% to 10,9%, respectively) [10]. Climbing numbers of overweight and obese women in our study could be explained by rapid nutritional transition, where changes in diet and lifestyle pattern through years contributed to over-nutrition, like other Latin American countries [32]. Growing economies in Latin America have been experiencing this transition, augmenting adult obesity rates explained by change in dietary pattern [33]: more intake of fats and carbohydrates (including raising consumption of refined foods), and less vegetables and fruits consumption. In addition, sedentary lifestyles and physical inactivity due to rapid urbanization have been contributing to escalating levels of over-nutrition [32].

As suggested by data of the Food and Agriculture Organization (FAO: http://faostat.fao.org/), Peruvian population increased their caloric intake during the studied years—from 2280 kcal/day in 1996 to 2563 kcal/day in 2009 (12% increase)—mainly coming from refine products, animal raw fat, vegetable fat and products rich in simple carbohydrates. Recent evidence suggests high prevalence of physical inactivity in Peruvian urban population [34], which added to inactive hobbies [35] and weight underestimation [36], among other factors, might have hastened the nutritional transition in Peru, starting in adult populations—supported by a recent nationally representative study of the Peruvian population, highlighting high levels of obesity in males and females between 30 to 59 years old [9].

Obesity and overweight vary within and between countries, reflecting social determinants in adult obesity burden [37]. Monteiro *et al*. [38], and Dinsa *et al*.[39] suggested that poor women start suffering from the burden of obesity when a nation's GNI reach $2500 and $1000, respectively.

According to the World Bank, Peru has accomplished a rapid economic development from 1996 to 2011, with a total GDP (Constant LCU) augmentation of 104.4% and GNI per capita (constant LCU) increase of 60.0% in this period. Moreover, Peru declined poverty levels from 58.7% in 2004 to 27.8% in 2011.

This rapid economic growth in Peru has brought little decrease in GNI index (e.g. 0.56 in 1998 and 0.48 in 2010), suggesting remaining inequalities. The relationship between SES and obesity changes according to which SES indicator is analyzed, and by place of residence; having that wealthier rural women seem to have more chance of being obese, with unclear association in the urban areas [40]. Conversely, Peruvian women with more education seem to have less odds of obesity, more noticeable in urban settings [40]. Further evidence should address when (if happened) obesity burden went towards people in less advantaged SES groups—including different SES indicators—in Peru; for understanding the effect of rapid national macroeconomic progress on obesity inequality.

On the other hand, Latin American countries showed declining rates of anemia, where their government entities have taken emphasized strategies to reduce this condition in children, including dietary supplements with ferrous sulfate and fortified foods [41], and even when this intervention might be dubiously effective in the short term [42], this could partially explain the decreasing trend of anemia percentage that we present in our manuscript for Peruvian children [13]. On the other hand, high levels of anemia have been reported in overweight or obese women from 3 developing countries (including Peru) [43], telling us that individuals from LMIC can be malnourished even if they appear in ranges of over-nutrition.

Remaining high levels of anemia in Peruvian children of preschool age and women of reproductive age could be explained by incomplete targeting of these vulnerable populations, mainly affecting children of rural settings [14]. Additional explanations may arise, such as the effect of Peruvian economic development on anemia, but stay beyond the objective of this manuscript. Further studies and interventions assessment should be performed to determine why Peruvian children and women still manifest current anemia figures.

Our study presents some limitations. First, it is a descriptive study and any apparent relationship interpreted from figures or tables between anthropometric measures variations and/or socio-demographic variables would only imply an ecologic association and readers should take this findings with precaution—since it could reflect an ecologic fallacy [44]. We must emphasize that our hypotheses regarding macroeconomic growth, change in diet, and/or enhancement of social programs on nutritional transition in Peru, represent also an ecological relation. In this manuscript we postulate that these factors seem the more plausible reasons behind our depicted trends. Formal studies assessing impact of interventions should be carried out, to evaluate nutritional programs among SES groups and settings in Peru. We were unable to evaluate the effect of other variables like wealth, diet or breastfeeding due to lack of comparability in measurement methodologies across waves or because unavailable data, nevertheless data from other sources, like FAO and World Bank, has been discussed in this section, for a better explanation of the probable causes of the trends.

On the other hand we should point-out that population-based surveys, used in this study, were representative of the entire Peruvian pre-school children and women in reproductive age (15–49 years) between1996 to 2011. We can assure with high certainty that our results depict the nutritional transition in Peru, which rapidly became an upper-middle income country in the last 20 years.

Our findings support the fact that Peru is on track to meet the Millennium Development Goals (MDG) (http://www.un.org/millenniumgoals), which include reduction of extreme poverty and hunger. However, Peru has been facing persistent malnutrition profiles, being over-nutrition dramatically escalating in adult women.

Even when under-nutritional indicators, in children under five have dropped, same as for overweight, and women's obesity have increased, the nutritional transition in Peru shows different patterns for urban and rural populations. Public policies should emphasize targeting both malnutrition conditions—under-nutrition/stunting, overweight/obesity and anemia—considering age and place of residence in rapid progressing societies like Peru.
