# Correction: Nutritional Transition in Children under Five Years and Women of Reproductive Age: A 15-Years Trend Analysis in Peru

**DOI:** 10.1371/journal.pone.0103356

**Published:** 2014-07-17

**Authors:** The PLOS ONE Staff

There is an error in reference 3. The correct reference is: WHO, UNICEF, World Bank (2012) Level & Trends in Child Malnutrition. UNICEF-WHO-The World Bank: Joint child malnutrition estimates - Levels and trends.

There is an error in reference 40. The correct reference is: Poterico JA, Stanojevic S, Ruiz-Grosso P, Bernabe-Ortiz A, Miranda JJ (2012) The association between socioeconomic status and obesity in Peruvian women. Obesity (Silver Spring) 20: 2283-2289.
